# Reviewer acknowledgement 2013

**DOI:** 10.1186/1471-2415-14-14

**Published:** 2014-02-13

**Authors:** Alice K Murray

**Affiliations:** 1 BioMed Central, 236 Gray's Inn Road, London WC1X 8HB, UK

## Abstract

**Contributing reviewers:**

The editors of BMC Ophthalmology would like to thank all of our reviewers who have contributed to the journal in Volume 13 (2013).

## 

Edoardo Abed

Italy

Marwan Abouammoh

Saudi Arabia

James Acheson

United Kingdom

James Acheson

United Kingdom

Roland Ackermann

Germany

Grazyna Adamus

USA

Martin-Paul Agbaga

USA

Rupesh Agrawal

Singapore

Frank Ahfat

United Kingdom

Seong Joon Ahn

South Korea

Ashish Ahuja

USA

Tan Aik Kah

Malaysia

Philip Alexander

United Kingdom

Jorge Alio

Spain

Fateme Alipour

Iran

Rana Altan-Yaycioglu

Turkey

Winfried Amoaku

United Kingdom

Winfried Amoaku

United Kingdom

Shantha Amrith

Singapore

James Aquavella

USA

Kaoru Araki-Sasaki

Japan

Navid Ardjomand

Austria

Nikolai Artemyev

USA

Alberto Artola

Spain

Francisco Ascaso

Spain

Abdulkabir Ayanniyi

Nigeria

Rajvardhan Azad

India

Seifollah Azadi

USA

Nigel Barnett

Australia

Ziad Bashshur

Lebanon

Jose Belda

Spain

Marc Biarnés

Spain

David Birch

USA

Alex Black

Australia

Mehmet Borazan

Turkey

Richard Bowman

United Kingdom

Antoine Brezin

France

Kathryn Burdon

Australia

Ben Burton

United Kingdom

Carlo Cagini

Italy

Antonio Calcagni

United Kingdom

Erika Camacho

USA

Math Candel

Netherlands

Gonzalo Carracedo

Spain

Heidi Cate

United Kingdom

Mauro Cellini

Italy

David Chan

Singapore

Errol Chan

Singapore

David Chang

USA

Raymond Chang

Hong Kong

Erik Chankiewitz

Germany

Jun Chen

China

Philip Chen

USA

Shih-Jen Chen

Taiwan

Wei Chen

China

Karen Chia

Singapore

Kyung Seek Choi

South Korea

Bruno Chrcanovic

Sweden

Jonathan Clarke

United Kingdom

Nicholas Colatrella

USA

Jon Martin Collinson

United Kingdom

David Crabb

United Kingdom

Nicolas Cuenca

Spain

Bin Cui

China

Phillippa Cumberland

United Kingdom

Annegret Dahlmann-Noor

United Kingdom

Sujata Das

India

Undurti Das

USA

Dawn Decarlo

USA

Sunil Deokule

USA

Eric Deshaies

USA

Efstathios Detorakis

Greece

Chuanqing Ding

USA

Ian Dooley

Ireland

Martin Duddy

United Kingdom

James Dunn

USA

Christoph Ehlken

Germany

Tom Eke

United Kingdom

Samer Elsherbiny

United Kingdom

Jennifer Evans

United Kingdom

Fernando Falcao-Reis

Portugal

Tarannum Fatima

India

Sascha Fauser

Germany

Antonio Fea

Italy

Consuelo Ferrer

Spain

Antonio Ferreras

Spain

Michele Figus

Italy

Paolo Fogagnolo

Italy

Farzin Forooghian

Canada

John Forrester

United Kingdom

Patrice Fort

USA

Frederick Fraunfelder

USA

Robert Gabriel

Hungary

Miriam Garcia-Fernandez

Spain

Santiago García-Lázaro

Spain

Gus Gazzard

United Kingdom

Asaad Ghanem

Egypt

Neha Goel

India

David Goldblum

Switzerland

Pablo Goldschmidt

France

Pablo Goldschmidt

France

Lan Gong

China

J Gonzalez-Meijome

Portugal

Lekha Gopal

Singapore

Iwona Grabska-Liberek

Poland

Karsten Gronert

USA

Ashok Grover

India

Astor Junior Grumann

Brazil

Venkata Gudlavalleti

India

Yan Guex-Crosier

Switzerland

Maged Habib

United Kingdom

Billy Hammond

USA

Inderraj Hanspal

United Kingdom

Martin Harris

United Kingdom

Sohan Hayreh

USA

Jiucheng He

USA

Owen Hee

Singapore

David Henson

United Kingdom

Takahiro Hiraoka

Japan

Cornelia Hirn

Switzerland

Sadao Hori

Japan

Hamid Hosseini

Iran

Marina Hovakimyan

Germany

Jacques Hugon

France

Rahat Husain

Singapore

Michele Iester

Italy

Akihiro Ikeda

USA

Makoto Inoue

Japan

Rafael Iribarren

Argentina

Murat Irkec

Turkmenistan

Hiroshi Ishikawa

USA

Takeshi Iwase

Japan

Aidila Jabbari

Malaysia

Nieraj Jain

USA

Vishal Jhanji

India

Davin Johnson

Canada

Jost Jonas

Germany

Sven Jonuscheit

United Kingdom

Jayaraman Kaliamurthy

India

Yogita Kanan

USA

Dimitrios Karagiannis

Greece

Satoshi Kashii

Japan

Anne Kasus-Jacobi

USA

Marzieh Katibeh

Iran

Costas Katsoulos

Greece

Ryo Kawasaki

Japan

Pearse Keane

United Kingdom

Fergal Kelleher

Australia

Kadircan H Keskinbora

Turkey

Jyoti Khadka

Australia

Naheed Khan

USA

Hemant Khanna

USA

Anthony Khawaja

United Kingdom

Chan Yun Kim

South Korea

Jeong Hun Kim

South Korea

Mee Kum Kim

South Korea

Bojana Kisic

Serbia

Yoshiaki Kiuchi

Japan

Guy Kleinmann

Israel

Thomas Kohnen

Germany

Daniel Kook

Germany

Bobby Korn

USA

Ajay Kumar Kotagiri

United Kingdom

Peter Koulen

USA

Vassilios Kozobolis

Greece

Ilse Krebs

Austria

Hatem Krema

Canada

Arun Kumar

United Kingdom

Matthias L&Uuml;Ke

Germany

Neil Lagali

Sweden

Timothy Lai

Hong Kong

Cedric Lamirel

France

Constantin Landes

Germany

Dennis Lanigan

Canada

Ruth Lapid-Gortzak

Netherlands

Eva Larsson

Sweden

Silvania Yuk Fan Lau

Hong Kong

John Lawrenson

United Kingdom

Llewellyn Lee

Singapore

Bin Li

China

Linjing Li

USA

Zhijie Li

USA

Qingfeng Liang

China

Yuanbo Liang

China

Shu Lang Liao

Taiwan

Hans Limburg

Netherlands

Rodrigo Lira

Brazil

Longqian Liu

China

Andrew Lloyd

United Kingdom

Alastair Lockwood

United Kingdom

Antonio Longo

Italy

Gang Luo

USA

Douglas Lyall

United Kingdom

Douglas Lyall

United Kingdom

Pir. Salim Mahar

Pakistan

Dimitra Makrynioti

Greece

Alexsandra Malaquias

Brazil

Miguel Maldonado

Spain

Baskaran Mani

Singapore

Gianluca Manni

Italy

Ahmad Mansour

Lebanon

Kaweh Mansouri

Switzerland

Enrico Martini

Italy

Guy Massry

USA

Wanjiku Mathenge

Rwanda

Hiroyuki Matsumoto

USA

Malcolm Mazow

USA

Martin McKibbin

United Kingdom

Charles McMonnies

Austria

Andrew McNaught

United Kingdom

Annal Meleth

USA

Marcel Menke

Switzerland

André Mermoud

Switzerland

Catherine Meyerle

USA

Zofia Michalewska

Poland

Dan Milea

Singapore

Q Mohamed

United Kingdom

Mehrdad Mohammadpour

Iran

Javier Montero

Spain

Majid Moshirfar

USA

Heather Moss

USA

Ahmed Mousa

Saudi Arabia

Jean-Claude Mwanza

USA

Taiji Nagaoka

Japan

Vinay Nangia

India

Giorgio Napolitano

Italy

Gabor Nemeth

Hungary

Irmingard Neuhann

Germany

Tsz Kin Ng

Hong Kong

Benjamin Nicholson

USA

Robert Nickells

USA

Esben Nielsen

Denmark

Nuwan Niyadurupola

United Kingdom

Winifred Nolan

United Kingdom

Hidetaka Noma

Japan

Mohammad Hosein Nowroozzadeh

Iran

Francesco Oddone

Italy

Seydi Okumus

Turkey

Sotaro Ooto

Japan

Eoin O'Sullivan

United Kingdom

Yanling Ouyang

Germany

Josephine Owoeye

USA

Manoj Parulekar

United Kingdom

Daksha Patel

United Kingdom

Sophia Pathai

United Kingdom

Jayter Paula

Brazil

Ryan Pedrigi

United Kingdom

Vijayalakshmi Perumalsamy

India

Sergio Petroni

Italy

Ioannis N. Petropoulos

United Kingdom

Gerald Pfeffer

United Kingdom

Amelie Pielen

Germany

David Pablo Pinero Llorens

Spain

Ana Belen Plaza

Spain

Eugenie Poh

Singapore

Philip Polkinghorne

New Zealand

Marianne Price

USA

María Puell

Spain

Victoria Pueyo

Spain

Claudio Punzo

USA

Sreekumari Pushpoth

United Kingdom

David Pye

Australia

Yi Qu

China

Visvanathan Ramamurthy

USA

Usha Raman

India

Andrew Ramsay

United Kingdom

Pradeep Ramulu

USA

Franziska G. Rauscher

Germany

Mohammad Reza Razeghinejad

Iran

Tony Realini

USA

Ian Rennie

United Kingdom

Alan Robin

USA

Luca Rossetti

Italy

Alan Rotchford

United Kingdom

Pierre-Raphael Rothschild

France

Barry Rovner

USA

Habiba Saedon

United Kingdom

Ahmed M. Saeed

Egypt

Wataru Saito

Japan

Ahmed Sallam

United Kingdom

Maria Teresa Sandinha

United Kingdom

Russell Saneto

USA

Krishnaiah Sannapaneni

India

Esin Sarý

Turkey

Tatsuhiko Sato

USA

Giacomo Savini

Italy

Suzie Scales

USA

Ronald Schuchard

USA

Michael Seider

USA

Sabyasachi Sengupta

India

Philip Severn

United Kingdom

Vijay Shanmuganathan

United Kingdom

Kei Shinoda

Japan

Dawn Sim

United Kingdom

Martin Sin

Czech Republic

Simon Skalicky

United Kingdom

Andrea Sodi

Italy

Neelam Sood

India

Torben Lykke Sorensen

Denmark

Chie Sotozono

Japan

Sanjay Srinivasan

Singapore

Oliver Stachs

Germany

Fiona Stapleton

Australia

David Steel

United Kingdom

Michael Stewart

USA

Mithu Storoni

Hong Kong

Michael Straiko

USA

Joel Sugar

USA

Young-Woo Suh

South Korea

David A Sullivan

USA

Yang Sun

USA

Kiyoshi Suzuma

Japan

Helen Swarbrick

Australia

Hiroki Takada

Japan

Yasuhiro Takahashi

Japan

Julia Talajic

Canada

Petrina Tan

Singapore

Junyan Tao

USA

Kristina Tarczy-Hornoch

USA

Hande Taylan Sekeroglu

Turkey

Allen Taylor

USA

Howard Tessler

USA

Peter Bjerre Toft

Denmark

Daniele Tognetto

Italy

Der-Chong Tsai

Taiwan

Fuu-Jen Tsai

Taiwan

Michael Tsatsos

United Kingdom

Takashi Ueta

Japan

Takashi Ueta

Japan

Michael Ulbig

Germany

Roxana Ursea

USA

Canan Utine

Turkey

Rasik Vajpayee

Australia

Tom Van Den Berg

Netherlands

Jeffrey Varanelli

USA

Praveen Vashist

India

Muthukkaruppan Veerappan

India

Alfredo Vega Estrada

Spain

Federico Velez

USA

Stephen Vincent

Australia

Setareh Vistamehr

USA

Anthony Vugler

United States Minor Outlying Islands

An-Guor Wang

Taiwan

Janey Wiggs

USA

Mark Willcox

Australia

Cathy Williams

United Kingdom

Lynne A Wolfe

USA

J Mario Wolosin

USA

Richard Wormald

United Kingdom

Edward Wylegala

Poland

Lixin Xie

China

Ling Yang

China

Paul Yang

USA

Shahin Yazdani

Iran

Tun K Yeo

Singapore

David Yorston

United Kingdom

Claus Zehetner

Austria

Dongfeng Zhang

China

Xiaojun Zhang

China

Yingfeng Zheng

China

Haogang Zhu

United Kingdom

Yehong Zhuo

China

## Pre-publication history

The pre-publication history for this paper can be accessed here:

http://www.biomedcentral.com/1471-2415/14/14/prepub

